# Prevalence and Clinical Features of Vestibular Migraine in Different Age Groups: Systematic Review and Meta-Analysis

**DOI:** 10.3390/neurosci7020037

**Published:** 2026-03-19

**Authors:** Lamees A. Alhajri, Renad S. Manez, Husna Irfan Thalib, Abdulelah F. Alshehri, Amjad M. Alramadan, Lama M. Alsulami, Mustafa A. Al Shankiti, Mahmoud Alhajji, Abdullah Almaqhawi

**Affiliations:** 1 College of Medicine, King Faisal University, Alahsa 31982, Saudi Arabia; amjadalramadan9931@gmail.com; 2 Medical Intern, College of Medicine, King Abdulaziz University, Jeddah 21589, Saudi Arabia; renadsaad16@gmail.com; 3 General Medicine Practice Program, Batterjee Medical College, Jeddah 21442, Saudi Arabia; husnairfan2905@gmail.com; 4 College of Medicine, Imam Mohammad Ibn Saud Islamic University (IMSIU), Riyadh 11646, Saudi Arabia; 5 College of Medicine, King Abdulaziz University, Jeddah 21589, Saudi Arabia; lamaalsulami1234@gmail.com; 6 College of Medicine, Umm Al-Qura University, Al-Qunfudhah 28821, Saudi Arabia; mustafa-alshankiti@hotmail.com; 7 Almoosa Specialist Hospital, Alahsa 36342, Saudi Arabia; mahajji@almoosahealth.com.sa; 8 Department of Family Medicine and Community, College of Medicine, King Faisal University, Alahsa 31982, Saudi Arabia; aalmuqahwi@kfu.edu.sa

**Keywords:** vestibular migraine, clinical features, prevalence, age-related differences, vertigo disorders

## Abstract

Introduction: Vestibular migraine (VM) is a frequent but underdiagnosed cause of episodic vertigo, characterized by vestibular symptoms often accompanied by migrainous features. Despite its relatively high prevalence, diagnosis remains clinically challenging and may differ depending on the diagnostic criteria used. This systematic review evaluates VM prevalence and clinical features across age groups to improve recognition and guide age-appropriate management. Methods: This systematic review and meta-analysis followed PRISMA guidelines and was registered in PROSPERO. This research was conducted using PubMed, Google Scholar, Cochrane, Web of Science, Wiley Online Library, and Embase. Two independent reviewers screened studies by title and abstract, and a separate pair screened full texts. Eligible studies were observational and reported prevalence or clinical features of VM. Results: A total of 874 publications were identified, leading to the review of 21 studies. Prevalence of VM varied widely, ranging from 6% to 35% in children and 2.7% to 40.9% in adults. Pooled prevalence across studies was 19% overall, 25% in children, and 14% in adults. Among patients with vertigo, the pooled prevalence was higher at 26%, with 33% in children and 18% in adults. Vertigo was the most consistent symptom in both age groups, and female predominance was observed in all age groups. Prevalence variability likely reflects diversity in applied diagnostic criteria and study design across included studies. Conclusion: VM is a common cause of vertigo, particularly in pediatric populations. Age-specific clinical features highlight the need for tailored diagnostic and management strategies. Future research should focus on large-scale prospective studies.

## 1. Introduction

Vestibular migraine (VM) is a subtype of migraine characterized by vestibular symptoms occurring in association with migrainous features [1]. It was previously known as migraine-associated vertigo, migraine-related vestibulopathy, and migrainous vertigo [2]. VM is increasingly recognized as the most common cause of episodic vertigo in both adult and pediatric populations [3]. Its clinical significance lies in its relatively high prevalence in the general population, which is estimated to be 1% over a lifetime and 0.9% over a year [4]. Additionally, VM accounts for about 7% of patients in dizziness clinics and 9% in migraine clinics [5]. Despite its commonality, it remains underdiagnosed, with only 20% of affected individuals receiving an accurate diagnosis [4,6].

The clinical presentation of VM typically includes spontaneous or positional vertigo, motion sensitivity, and imbalance. These symptoms are often accompanied by migraine features such as photophobia, phonophobia, nausea, aural disturbances, and headache [1,2,3]. Attacks usually last from several minutes to 72 h [1,2,3]. However, diagnosing VM is often challenging, as it remains a clinical diagnosis based primarily on patient history and symptom patterns [1,2,7]. There are no definitive laboratory or imaging tests to confirm VM, and its wide range of symptoms frequently overlaps with other vestibular disorders, such as Ménière’s disease and benign paroxysmal positional vertigo, making it challenging to distinguish in routine clinical practice [2,3,4,5]. These diagnostic challenges contribute to delayed or missed diagnoses, which have negative effects on treatment outcomes and patient quality of life. Additionally, accurate diagnosis is further complicated by the fact that different age groups and clinical contexts may have different interpretations and applications of VM diagnostic criteria.

VM affects individuals across all age groups, but its presentation can vary notably by age. Adults usually experience well-defined migrainous features, such as pulsatile headache, photophobia, phonophobia, and aura, occurring alongside episodes of vertigo or imbalance [3]. In contrast, children with VM more frequently experience episodic vertigo, unsteadiness, and motion sensitivity, while classical migraine symptoms may be absent or less apparent, complicating the diagnosis further [8,9,10]. These variations demonstrate how clinical features of VM vary significantly with age, which can complicate recognition and delay appropriate management. Additionally, although gender differences in VM prevalence and presentation have been reported, they remain poorly understood, especially within specific age groups.

Even though VM has gained more attention in recent years, significant gaps remain in understanding its actual prevalence and age-related clinical manifestations [6,8,9]. Most of the current studies focus on adults, while studies on children and adolescents are limited [8]. Therefore, this systematic review primarily aims to evaluate the prevalence and clinical presentations of vestibular migraine across different age groups, while also assessing applied diagnostic criteria and potential gender-related differences as secondary objectives. By systematically examining these aspects, the review aims to clarify age-specific patterns of VM, highlight diagnostic challenges, and provide insights to enhance recognition, diagnosis, and management across the lifespan.

## 2. Materials and Methods

### 2.1. Protocol and Registration

This systematic review and meta-analysis were conducted in accordance with the PRISMA 2020 guidelines. The study protocol was prospectively registered in PROSPERO under the registration number CRD420251066524.

### 2.2. Data Sources and Search Strategy

A comprehensive literature search was conducted across PubMed/MEDLINE, Google Scholar, Cochrane/CENTRAL, Web of Science, Wiley Online Library, and Embase/Ovid, encompassing the period from database inception through 10 June 2025. The search method integrated MeSH terms and keywords: (“vestibular migraine”) AND (“prevalence” OR “epidemiology” OR “frequency” OR “clinical features” OR “symptoms” OR “signs” OR “manifestations”) AND (“age groups” OR “children” OR “pediatric” OR “adolescents” OR “adults” OR “elderly” OR “older adults”). Each database was searched using a specific search syntax created for each one, incorporating the relevant keywords. The Google Scholar results were confined to the initial 20 pages ordered by relevance to guarantee reproducibility. All search results were imported into the Rayyan software 1.4.3 for management and screening, and duplicates were removed through both automated and manual verification.

### 2.3. Eligibility Criteria

Studies were deemed eligible if they included patients of all age groups, encompassing both pediatric and adult populations, diagnosed with vestibular migraine. Eligible study designs encompassed observational studies (cross-sectional, cohort, or case–control) and case series with retrievable data. Studies were mandated to report either prevalence estimates or detailed clinical features of vestibular migraine. As the definition of pediatric and adult age groups varies across studies, a clearly defined age range was required for inclusion. The reported age range for each study is presented in the tables in the results section to ensure clarity and transparency. The diagnostic criteria used in each study were not required for inclusion; as they differed across studies, they were recorded during data extraction and considered when analyzing prevalence and clinical features.

Studies were excluded if the age group was not explicitly specified, if they addressed other vestibular or migraine disorders without a definitive diagnosis of vestibular migraine, or if they were conference abstracts, reviews, editorials, case reports, animal studies, or non-peer-reviewed publications.

### 2.4. Study Selection Process

Following the removal of duplicates, two independent reviewers screened the titles and abstracts of all retrieved records based on the eligibility criteria. The articles that passed this initial screening were then subjected to full-text review by two other reviewers. Any disagreements were resolved through discussion.

### 2.5. Data Extraction and Management

Two authors independently extracted data into a standardized sheet. Separate sheets were used for prevalence data and clinical features, which are reported and analyzed separately in the results section to ensure a clear and organized presentation. Extracted variables consisted of study characteristics (author, country, setting, study design, and year of publication), participant demographics (age group studied, age range, gender distribution), diagnostic criteria employed, familial history of migraine, clinical features, most common clinical feature reported, episode duration, and prevalence estimates. Additional variables were extracted but not analyzed in the results section due to insufficient reporting across studies; these included age of onset for vertigo and migraine, vestibular signs identified during clinical examination, and frequency of episodes. Data was extracted and, where possible, stratified by age group to allow for comparisons across different populations.

### 2.6. Risk of Bias Assessment

Two independent reviewers assessed the methodological quality using the Joanna Briggs Institute (JBI) Critical Appraisal Checklists for analytical cross-sectional studies, case series, and case–control studies, while the Newcastle–Ottawa Scale (NOS) was used for cohort and case–control designs. Domains included sample representativeness, clarity of inclusion criteria, validity and reliability of exposure and outcome measurements, identification and management of confounding factors, and appropriateness of statistical analysis. Studies with three or more domains rated as “No” or “Unclear” in the JBI assessment, or with NOS scores below six, were categorized as high risk of bias, flagged for sensitivity analyses, and, where applicable, excluded from pooled estimates.

### 2.7. Statistical Analysis

Quantitative synthesis was performed using Stata/BE v17.0 (StataCorp, College Station, TX, USA) as the primary software, consistent with the protocol’s allowance for RevMan or other meta-analysis software. Pooled prevalence estimates were calculated using a random-effects model (DerSimonian and Laird method) to account for between-study variability. As a robustness check, the restricted maximum likelihood (REML) estimator was also applied, yielding consistent results. Age-stratified data from individual studies were analyzed as independent units, and robust variance estimation was used in sensitivity analyses to address potential clustering within studies.

Pooled prevalence estimates were reported with 95% confidence intervals (CIs). Continuous variables, such as age of onset and frequency of episodes, were summarized as means and standard deviations (SDs), with conversions from medians and interquartile ranges performed using validated statistical methods. Heterogeneity was quantified using the I^2^ statistic, with thresholds of 25%, 50%, and 75% representing low, moderate, and high heterogeneity, respectively.

In cases of substantial heterogeneity (I^2^ > 75%), subgroup analyses were performed by age group, geographic location, diagnostic criteria, and study design. Publication bias was evaluated using Egger’s regression test and visual inspection of funnel plots when at least 10 studies were available. Sensitivity analyses were performed by excluding studies at high risk of bias.

All statistical tests were two-tailed, with *p*-values < 0.05 considered statistically significant. Forest plots were generated to visually present both individual study estimates and pooled estimates.

## 3. Results

### 3.1. Included Studies

**Figure 1:**
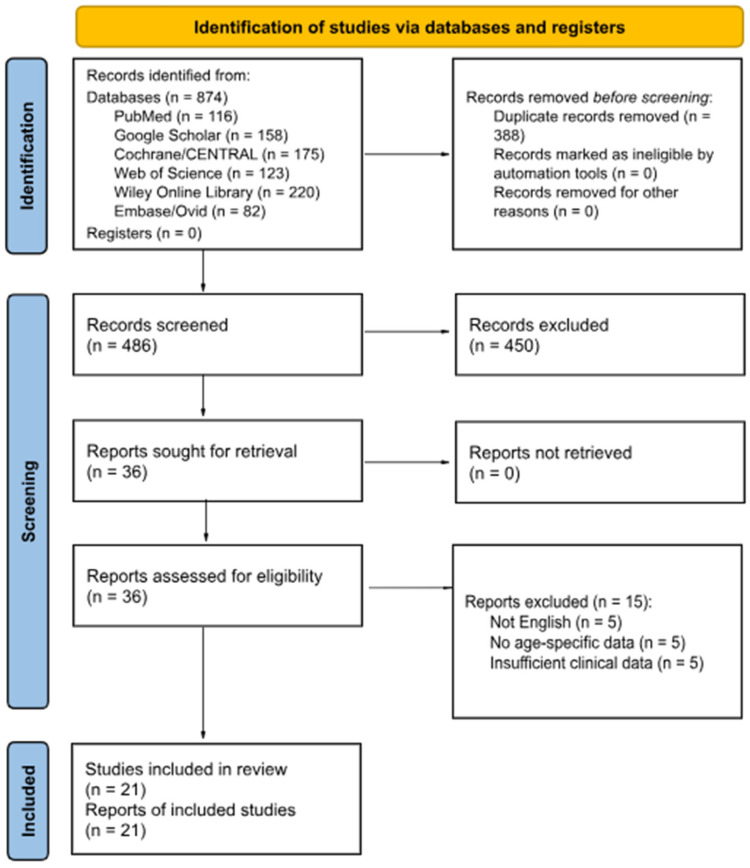
PRISMA Flow Diagram of Study Identification and Screening.

A total of 874 records were identified through database searches, including PubMed, Google Scholar, Cochrane, Web of Science, Wiley Online Library, and Embase. After removing 388 duplicates, 486 records were screened by title and abstract. Of these, 450 records were excluded due to irrelevance to the research question. The remaining 36 full-text articles were assessed for eligibility. Following full-text review, 15 studies were excluded due to non-English text, lack of age-specific data, or insufficient clinical information related to vestibular migraine. Finally, 21 studies met the inclusion criteria and were included in the systematic review (Figure 1).

### 3.2. General Characteristics of Included Studies

Table 1 provides an overview of the general methodological characteristics of the included studies. The publication timeline spans from 2015 to 2025, which reflects nearly a decade of research in the field of vestibular migraine (VM). Most studies were conducted in tertiary hospitals or subspecialty outpatient clinic settings, thereby highlighting that VM is largely studied in specialized care environments rather than in general community populations. The geographical distribution is wide-ranging, encompassing Asia (Malaysia, South Korea, Turkey, China, Japan, India, and the UAE), Europe (Germany, Italy, Spain), North America (the USA), South America (Argentina), and Australia. This ensures a highly specialized perspective; however, variations in diagnostic criteria and clinical pathways across healthcare systems may influence outcomes.

In terms of methodology, retrospective designs (five studies) were often conducted using hospital chart reviews and institutional records to characterize VM cohorts. Cross-sectional studies (seven in total, including multicenter efforts) are also common, which aim to capture the clinical phenotype, prevalence, and comorbidities of VM across diverse outpatient populations. The inclusion of multicenter studies (e.g., Soo-Jin Cho, 2015 [11]; Çelebisoy et al., 2022 [12]; Teggi et al., 2018 [13]) enhances external validity by including broader, more heterogeneous patient samples.

**Table 1:** Study Characteristics of Included Studies. Abbreviations: UMMC: University of Malaya Medical Center; ICHD-3: International Classification of Headache Disorders, 3rd edition; ENT: Ear, Nose and Throat; VM: Vestibular Migraine; UAE: United Arab Emirates, USA: United States of America; UK: United Kingdom.

Study ID (Author, Year)	Study Title	Country	Setting	Study Design	Year of Publication
Jeyasakthy Saniasiaya, 2024 [14]	Experience from the First Pediatric Vestibular and Balance Clinic in a Multiracial Asian Setting	Malaysia (UMMC), in collaboration with the UK (Alder Hey Hospital)	Tertiary hospital (Pediatric Vestibular and Balance Clinic)	Retrospective	2024
Zülal Özdemir Uslu, 2024 [15]	Childhood vertigo: A retrospective series of 791 cases	Turkey	Pediatric neurology outpatient clinic, Ankara Keçiören Training and Research Hospital	Retrospective	2024
Soo-Jin Cho, 2015 [11]	Vestibular migraine in multicenter neurology clinics according to ICHD-3 beta appendix criteria	South Korea	Multicenter neurology outpatient departments (11 hospitals)	Cross-sectional	2015
Thyra Langhagen, 2015 [8]	Vestibular migraine in children and adolescents: clinical findings and laboratory tests	Germany	German Center for Vertigo and Balance Disorders	Retrospective	2015
Teggi et al., 2018 [13]	Clinical Features of Headache in Patients With Vestibular Migraine: The VM-Phenotypes Projects	Italy & Spain	Multicenter outpatient clinics	Cross-sectional	2018
Gedik-Soyuyuce et al., 2021 [16]	Vestibular disorders in children: a retrospective analysis	Turkey	Tertiary hospital (Vertigo Center)	Retrospective	2021
Lin & Rauch, 2024 [17]	Current Demography and Treatment Strategy of Vestibular Migraine in Neurotologic Perspective	USA	Otolaryngology—Head & Neck Surgery clinics	Retrospective multicenter	2024
Çelebisoy et al., 2022 [12]	Vestibular migraine, demographic and clinical features of 415 patients	Turkey	8 tertiary neurology clinics	Cross-sectional, multicenter	2022
Power et al., 2018 [18]	Clinical characteristics and treatment choice in vestibular migraine	Australia	Tertiary balance disorders clinic	Cross-sectional study	2018
Dessai et al., 2019 [19]	Prevalence of Vestibular Migraine in Dubai	UAE	Outpatient otorhinolaryngology clinic	Prospective observational study	2019
Teggi et al., 2018 [13]	Clinical Features, Familial History, and Migraine Precursors in VM patients	Italy & Spain	ENT outpatient and vertigo clinics	Cross-sectional multicenter	2018
Toriyama et al., 2021 [20]	Clinical features of definite vestibular migraine through central sensitization	Japan	Headache outpatient clinic (Shinshu University)	Cross-sectional study	2021
Swain et al., 2023 [21]	Vertigo in pediatric age group: Our experiences	India	Tertiary care teaching hospital (ENT department)	Retrospective	2023
Carmona et al., 2018 [22]	Vestibular Pathology in Pediatric Population: Relevance of VM	Argentina	Pediatric tertiary-care hospital (ENT Department)	Retrospective, observational, cross-sectional	2018
Zhang et al., 2015 [23]	ICHD-3 beta field testing of vestibular migraine in China	China	Neurology outpatient department, tertiary hospital	Retrospective observational	2015
Alicia Wang, 2020 [24]	Multifactorial Characteristics of Pediatric Dizziness and Imbalance	USA	Database of all patients seen at a pediatric vestibular program	Retrospective cohort study	2020
Juliana Duarte, 2020 [25]	Vestibular Syndromes in Childhood and Adolescence	Brazil	Pediatric neurotology clinic (2010–2019)	Cross-sectional study	2020
Hyo-Jung Kim, 2020 [26]	Etiologic distribution of dizziness and vertigo in a referral-based dizziness clinic in South Korea	South Korea	Seoul National University Bundang Hospital, referral-based dizziness clinic	Retrospective	2020
Alana L. Ferreira, 2024 [27]	Dizziness and Imbalance Across the Lifespan: Findings of a Pediatric and Adult Vestibular Clinic	USA	Electronic medical records of pediatric & adult tertiary care hospitals (Philadelphia, Bronx)	Retrospective chart review	2025
Eric J. Formeister, 2018 [28]	The Epidemiology of Vestibular Migraine: A Population-based Survey Study	USA	Nationwide household survey (NHIS)	Population-based cross-sectional survey	2018
Lee et al., 2017 [29]	Prevalence of vestibular and balance disorders in children and adolescents according to age: A multi-center study	South Korea	Multi-center hospitals (11 otolaryngology departments)	Retrospective chart review, multi-center	2017

### 3.3. Clinical Features of Vestibular Migraine

In total, 15 studies reported clinical features, including both pediatric (6 studies), as described in Table 2, and adult (9 studies), as described in Table 3. This highlights that VM is diagnosed across the lifespan. The sex distribution indicates female predominance across all studies.

Six pediatric studies included children from one year of age up to eighteen years (Table 2). Diagnostic criteria were mainly based on the International Classification of Headache Disorders (ICHD-3) and the Bárány Society definitions, with some pediatric adaptations. Family history of migraine was frequent, ranging from more than sixty percent to nearly ninety percent of patients. Clinical features described in these cohorts included vertigo, headache, nausea, vomiting, photophobia, phonophobia, motion sickness, tinnitus, and, in some cases, developmental issues such as speech or balance delay. Motion sickness and headache were often highlighted, but vertigo was consistently reported across all studies and represented the most common clinical feature. Episode duration varied widely, from very short attacks lasting seconds to prolonged episodes lasting hours or days. All studies showed female predominance, indicating that the gender pattern of vestibular migraine is present from childhood.

Nine adult studies covered patients from late adolescence through older age (Table 3). The diagnostic criteria used were predominantly ICHD-3 and Bárány Society criteria, which are broadly compatible and conceptually aligned, as the Bárány criteria were developed in collaboration with the International Headache Society (IHS) and later incorporated into ICHD-3 [2]. A positive family history of migraine was common and was reported in between one-fifth and almost ninety percent of cases, with several studies noting clustering in first-degree relatives. The clinical spectrum was broad but consistent, with vertigo reported in almost all patients. Other frequent features were nausea, headache, vomiting, photophobia, phonophobia, tinnitus, aural fullness, and motion sensitivity. Less common findings included visual aura, syncope, and cutaneous allodynia. Vertigo and nausea were most often identified as the predominant clinical features. The duration of episodes varied considerably, from seconds to minutes in some cases to hours and several days in others, with most within the 5 min to 72 h range defined by current criteria. All adult studies reported female predominance.

### 3.4. Prevalence of Vestibular Migraine (VM) Among the General Population

The prevalence of vestibular migraine (VM) in pediatric populations varied substantially, ranging from 6.1% to 35.0% (Table 4). The lowest estimate was reported by Carmona et al. (2018) [22], with only 6.1% of 247 children meeting criteria for VM, while the highest values were observed in Alicia Wang (2020) [24] and Hyo-Jung Kim (2020) [26], both reporting prevalence rates of approximately 35%. Several studies reported intermediate estimates. Swain et al. (2023) [21] found a prevalence of 30.8% in a smaller cohort, while Lee (2017) [29] reported 29.2%. Alana Ferreira (2024) [27] and Gedik-Soyuyuce et al. (2021) [16] described similar values, with VM affecting 23–24% of pediatric patients in their clinical cohorts. Juliana Duarte (2020) [25] reported a more modest prevalence of 16.2%.

The prevalence of vestibular migraine (VM) among adult patients demonstrated wide variability, ranging from 2.7% to 40.9% (Table 5). The lowest prevalence was reported by Formeister (2018) [28] in a very large U.S. cohort of over 21,000 patients, where only 2.7% were diagnosed with VM. Similarly, relatively low estimates were reported by Cho (2015) [11] in South Korea (4.6%) and Alana Ferreira (2024) [27] in Brazil (4.7%). Hyo-Jung Kim (2020) [26] found a somewhat higher prevalence of 11.5% in a large Korean sample of more than 20,000 patients. At the other end of the spectrum, smaller clinic-based studies reported much higher prevalence values. Power et al. (2018) [18] found VM in 40.9% of 220 patients, while Toriyama et al. (2021) [20] reported a prevalence of 26.5% and Dessai et al. (2019) [19] described 12.8%. Lin & Rauch (2024) [17] also reported a relatively high rate of 15.9% in their U.S. cohort.

### 3.5. Prevalence Meta-Analysis of Vestibular Migraine (VM) Among the General Population

**Figure 2:**
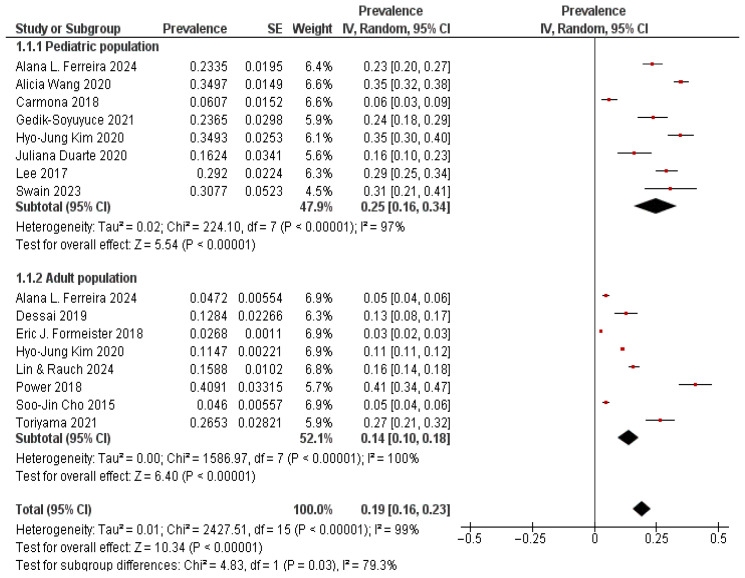
Forest Plot 1: Prevalence of Vestibular Migraine among the general population; Subgroup analysis by age group [11,16,17,18,19,20,21,22,24,25,26,27,28,29].

The pooled prevalence of vestibular migraine across all included studies was 19% (95% CI: 16–23%), though there was very high heterogeneity (I^2^ = 99%, *p* < 0.00001) (Figure 2). When stratified by age group, the prevalence was notably higher in the pediatric population, with a pooled estimate of 25% (95% CI: 16–34%) compared to 14% (95% CI: 10–18%) in adults. The difference between subgroups was statistically significant (*p* = 0.03). Among children, prevalence estimates varied from as low as 6% (Carmona, 2018 [22]) to as high as 35% (Alicia Wang, 2020 [24]; Hyo-Jung Kim, 2020 [26]). Most clinic-based cohorts reported values between 20 and 30%.

In adults, lower prevalence was observed in large population-level studies such as Formeister (2018) [28] and Cho (2015) [11] (2–5%). Much higher rates were seen in smaller specialty cohorts such as Power (2018) [18] (41%) and Toriyama (2021) [20] (27%). Overall, the analysis highlights that VM is more frequently identified in pediatric patients with vertigo compared to adults; however, prevalence values vary widely depending on study design and clinical setting.

**Figure 3:**
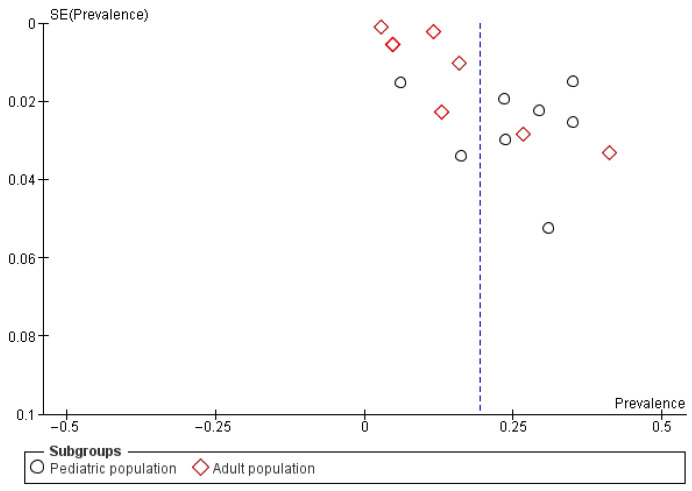
Funnel Plot 1: Prevalence of Vestibular Migraine among the general population; Subgroup analysis by age group.

The funnel plot for studies reporting vestibular migraine (VM) prevalence shows asymmetry. This suggests potential publication bias or small-study effects (Figure 3). Pediatric studies (circles) and adult studies (diamonds) are both represented. Several smaller studies with higher prevalence estimates are scattered away from the pooled effect line. Large population-based cohorts cluster closer to the lower prevalence values. This distribution indicates that smaller clinical studies may overestimate prevalence compared to larger multicenter or community-based cohorts.

### 3.6. Prevalence of Vestibular Migraine (VM) Among Vertigo Patients

The prevalence of vestibular migraine (VM) among patients presenting with vertigo showed marked variability across studies (Table 6), ranging from 11.9% to 40.9%. The lowest estimate was reported by Hyo-Jung Kim (2020) [26] in a very large Korean cohort of over 21,000 patients, where 11.9% were diagnosed with VM. In contrast, Gedik-Soyuyuce et al. (2021) [16] found the highest prevalence, with 40.9% of 203 patients affected. Several other studies reported prevalence values in the range of 23–35%, including Alicia Wang (2020) [24] with 34.9%, Formeister (2018) [28] with 23.5%, and Lee (2017) [29] with 29.2%. Smaller clinic-based cohorts, such as Juliana Duarte (2020) [25] and Swain et al. (2023) [21], also reported relatively high rates of around 30%. Meanwhile, moderate estimates were seen in Lin & Rauch (2024) [17] at 15.9% and Dessai et al. (2019) [19] at 12.8%.

### 3.7. Prevalence Meta-Analysis of Vestibular Migraine (VM) Among Vertigo Patients

**Figure 4:**
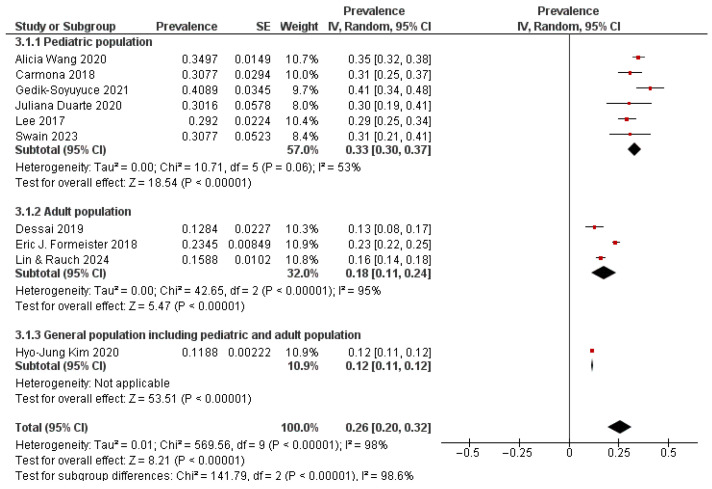
Forest Plot 2: Prevalence of Vestibular Migraine among vertigo patients; Subgroup analysis by age group [16,17,19,21,22,24,25,26,28,29].

Subgroup analysis of patients presenting with vertigo showed that the pooled prevalence of vestibular migraine (VM) was 33% (95% CI: 30–37%) in pediatric cohorts and 18% (95% CI: 11–24%) in adult cohorts (Figure 4). A large mixed-age general population study reported a lower prevalence of 12% (95% CI: 11–12%). Heterogeneity was moderate among pediatric studies (I^2^ = 53%) but high among adult studies (I^2^ = 95%). This reflects variability across clinical settings and sample sizes. Overall, the pooled prevalence of VM among vertigo patients across all groups was 26% (95% CI: 20–32%), with a statistically significant difference between subgroups (*p* < 0.00001). These findings indicate that VM is diagnosed more frequently in children compared to adults. Prevalence estimates from unselected general populations remain lower (12%).

**Figure 5:**
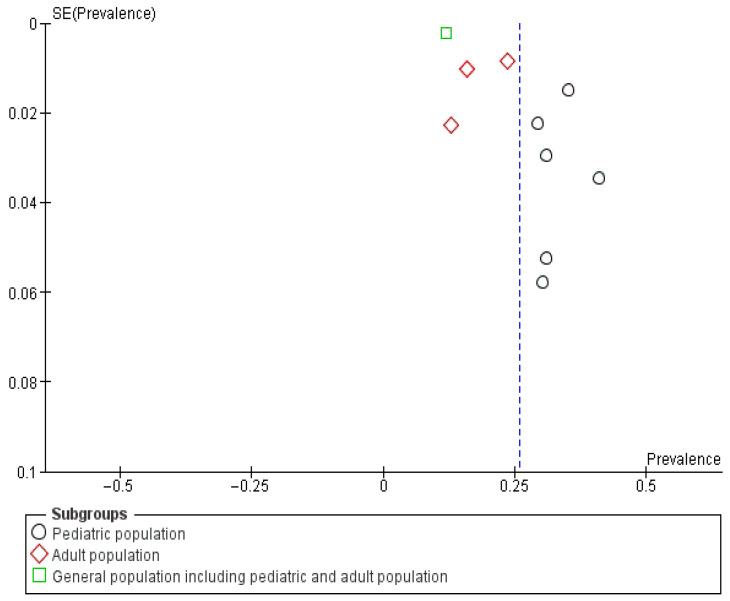
Funnel Plot 2: Prevalence of Vestibular Migraine among vertigo patients; Subgroup analysis by age group.

The funnel plot assessing publication bias in studies of vestibular migraine (VM) prevalence among vertigo patients showed mild asymmetry across subgroups (Figure 5). Pediatric studies (circles) and adult studies (diamonds) were distributed more widely. Smaller studies tend to report higher prevalence estimates. The single large general population study (green square) clustered tightly at a lower prevalence. Overall, the distribution suggests potential small-study effects, particularly in pediatric and adult clinical cohorts.

### 3.8. Outcomes, Strengths, and Limitations of Included Studies

The main statistical findings and methodological evaluations of the studies showed varied outcomes. In terms of focus, eight studies reported both prevalence and clinical features, while the remainder emphasized either prevalence alone or purely clinical characterizations. Statistical analysis was inconsistently reported: although 14 studies listed some statistical outputs (*p*-values, odds ratios, regression models), most did not provide detailed multivariable adjustments. Only a minority of the studies specifically reported confounder adjustments (e.g., accounting for comorbid chronic migraine or demographic stratification). This limits causal inference and highlights the predominance of descriptive research rather than analytical research in the field.

Strengths consistently included large sample sizes, multicenter collaborations, standardized diagnostic criteria, and structured clinical assessments. These elements improve reliability and generalizability. Conversely, the most common limitations were retrospective study design, referral bias from tertiary centers, lack of control groups, absence of long-term follow-up, and incomplete audiological/vestibular testing in children. Nearly all studies declared no conflicts of interest, suggesting a low risk of bias from funding sources. However, the studies demonstrate that VM is now identified as a leading cause of vertigo in both adult and pediatric populations. However, the reliance on hospital-based, retrospective cohorts and the lack of prospective controlled studies indicate that critical findings about longitudinal prognosis, treatment response, and real-world prevalence require further clinical research.

### 3.9. Risk of Bias Assessment

**Figure 6:**
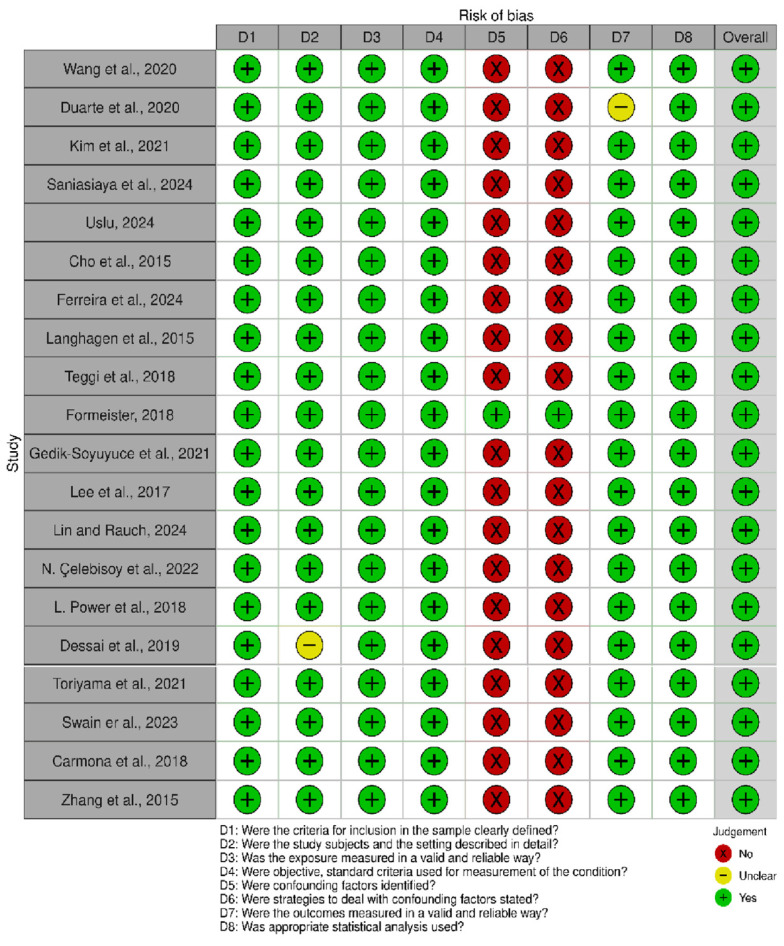
Risk of bias traffic plot for included studies assessed using the JBI tool across eight domains [11,12,13,14,15,16,17,18,19,20,21,23,24,25,26,27,28,29].

Risk of bias assessment using the Joanna Briggs Institute (JBI) tool showed that most studies were rated low risk across domains related to inclusion criteria, study setting, exposure measurement, outcome assessment, and statistical analysis (Figure 6). However, nearly all studies were judged to have a high risk of bias for confounding, as only a few studies identified or adequately addressed potential confounders. One study (Dessai, 2019 [19]) had some concerns regarding clarity of subject description, and another (Duarte, 2020 [25]) had minor concerns regarding outcome reliability. Despite these limitations, the overall risk of bias was considered low for all included studies.

**Figure 7:**
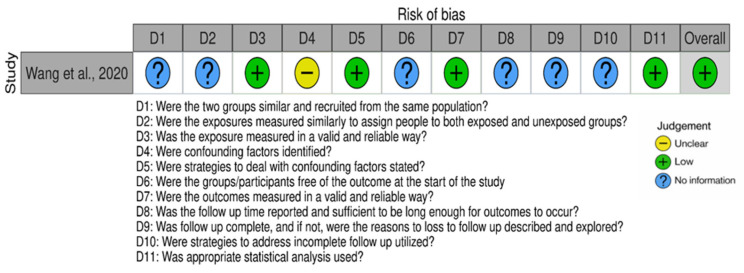
Risk of bias traffic plot for included studies assessed using the NOS tool across eleven domains [24].

Assessment of the cohort study using the Newcastle–Ottawa Scale (NOS) indicated an overall low risk of bias (Figure 7). Most domains, including exposure measurement, outcome assessment, and statistical analysis, were rated as low risk. However, several items, such as group comparability, baseline outcome status, and follow-up details, were judged as unclear or not reported. This reflects incomplete methodological information. Despite these gaps, the study was considered to be of acceptable methodological quality with an overall low risk of bias.

## 4. Discussion

This systematic review and meta-analysis confirm that vestibular migraine (VM) is both highly prevalent and variable in its clinical manifestation across the lifespan. By synthesizing evidence from 21 studies conducted between 2015 and 2025, this review shows that age influences both prevalence and clinical features.

### 4.1. Clinical Features in Pediatric and Adult Populations

Based on our analysis, VM presents with overlapping, but not identical, clinical features in pediatrics and adults.

In pediatric patients, vertigo is the most consistent symptom accompanied by headache, nausea, vomiting, photophobia, phonophobia, tinnitus, and motion sickness. Thus, the pediatric population typically demonstrates both vertiginous and migrainous symptoms, while otological features, most notably tinnitus, are also frequently reported. The variability in duration of episodes from only a few seconds to several hours or even days complicates the diagnosis in pediatrics, as short episodes may be easily misattributed to other conditions. A strong family history of migraine is observed, with reported rates averaging as high as 60–90% [8,22]. When a positive family history is present, vestibular migraine should be in the top differential diagnosis of pediatric vertigo. Furthermore, the variation in diagnostic criteria applied across studies, most commonly ICHD-3 and Bárány Society criteria with pediatric adaptations, likely contributes to heterogeneity in the reported prevalence and clinical spectrum. The adaptations made for pediatric populations may not be uniformly applied, leading to discrepancies that complicate research and clinical practice. These findings emphasize the need for standardized, pediatric-specific criteria to ensure early recognition and diagnostic consistency, The lack of universally accepted pediatric-specific diagnostic criteria likely contributes to both heterogeneity across studies and underdiagnosis in clinical practice.

Furthermore, the idea of the “vestibular march” has been reported in pediatric populations, indicating that benign paroxysmal vertigo of childhood (BPVC) may be an early migraine spectrum manifestation that develops into vestibular migraine in adolescence or adulthood. The idea that vestibular migraine may occur along a clinical continuum, starting in early childhood with episodic vertigo syndromes and gradually evolving into more distinct migraineous vestibular episodes, is supported by this developmental pattern. Understanding this trend is especially crucial for long-term monitoring and early diagnosis [30].

Adults, in contrast, show a more consistent symptom profile. They exhibit near-universal vertigo, nausea, vomiting, headache, photophobia, phonophobia, tinnitus, aural fullness, and motion sensitivity, with vertigo and nausea emerging as the predominant features. Thus, adults also experience vertiginous, migrainous, and otological symptoms. This is consistent with prior research, where otological symptoms have been strongly associated with VM, with tinnitus identified as the most common manifestation [31]. Episode duration is more uniform and mostly ranges from 5 min to 72 h in line with ICHD-3 criteria, in contrast to the broader variability observed in pediatrics. Even though a positive family history of migraine is also common in adults, reported rates vary more widely (approximately 20–90%) compared with children, suggesting that genetic predisposition may be more consistently expressed early in life [13,18]. Most adult studies utilize the ICHD-3 and Bárány Society criteria; however, significant diagnostic inconsistencies persist, as some studies apply only one criterion, while others apply both. It is crucial to highlight that the Bárány Society’s initial diagnostic criteria, established in 2012, were developed in conjunction with the International Headache Society and then adopted into the International Classification of Headache Disorders, 3rd edition (ICHD-3). As a result, these criteria are broadly consistent and theoretically aligned, however modest changes in application between research may lead to heterogeneity [32]. This reflects the complexity of vestibular migraine and the absence of a single accepted diagnostic criterion able to capture all cases. The most updated diagnostic criteria were jointly developed by the Bárány Society and ICHD-3 and published in 2022 [32]. This version incorporated only literature updates, while the original criteria from 2012 remained unchanged [32]. It diagnosis VM in patients who experience at least five episodes of moderate-to-severe vestibular symptoms lasting 5 to 72 h, with at least half of the episodes accompanied by migraine features such as headache, photophobia and phonophobia, or visual aura, in the setting of a current or past history of migraine, provided that the symptoms cannot be more appropriately explained by another condition [32].

Both pediatric and adult populations show a marked female predominance, a finding well documented in the literature, suggesting that the gender pattern of vestibular migraine is established in childhood [32]. This genetic predisposition has been proposed to be due to an autosomal dominant pattern of inheritance with lower penetrance in men, which explains the female predominance [33]. The shared combination of vertiginous and migrainous symptoms, along with familial clustering and gender distribution, supports the view of VM as a clinical continuum rather than distinct pediatric and adult entities.

### 4.2. Prevalence of VM Across the General Population

The pooled prevalence of VM across all age groups was 19% (95% CI: 16–23%). These rates are significantly higher than the commonly cited prevalence of 1–3% in the general population [7,34]. This high prevalence may be attributed to increased recognition of VM and increased application of ICHD-3/Bárány criteria in recent years. Stratified analysis revealed a significantly greater prevalence in pediatrics, 25% (95% CI: 16–34%), than in adults, 14% (95% CI: 10–18%) (*p* = 0.03). This age-related difference is consistent with the findings of Ori et al., who reported that the diagnosis of definite vestibular migraine decreases with advancing age [35]. It is important to note that study settings have a significant impact on VM prevalence: estimates from community-based cohorts are low, whereas those from referral and clinic-based studies are significantly higher, which is in line with previous research [7]. For instance, a large U.S. population survey identified a prevalence of only 2.7% [28], whereas a specialized clinic cohort reported 40.9% [18].

### 4.3. Prevalence of VM Among Vertigo Patients

When restricted to vertigo populations, VM accounted for 33% of pediatric cases and 18% of adult cases. Combined, our pooled prevalence of 26% confirms that VM represents a common symptomatology of recurrent vertigo across all age groups, particularly in children, where up to one-third of vertigo cases may be attributable to VM. In fact, multiple studies indicated that VM is the most common cause of vertigo [36]. Other important differential diagnoses of vertigo include Ménière’s disease, benign paroxysmal positional vertigo (BPPV), vestibular neuritis, vascular vertigo, persistent perceptual postural dizziness (PPPD), primary dysautonomia, migraine with brainstem aura, transient ischemic attack, and episodic type 2 ataxia. Knowing these differential diagnoses is important, as they often present with overlapping clinical features that make the distinction between them difficult. In particular, several studies have reported that differentiating VM from Ménière’s disease is challenging [37]. This is evident in a retrospective study, which demonstrated that 13% of patients met the criteria for both VM and Ménière’s disease [38]. Misdiagnosis is therefore common, with 51.5% of VM patients previously diagnosed as having Ménière’s disease [39]. These findings necessitate careful diagnostic evaluation of vertigo disorders, and refinement of diagnostic criteria remains imperative.

### 4.4. Interpretation and Mechanisms

The higher prevalence rates of VM in pediatrics could stem from referral bias from specialized clinics, increased sensitivity to vestibular and sensory triggers, and strong genetic predisposition, as a positive family history is reported in the majority of cases. On the other hand, in adults, the lower prevalence rates could stem from under recognition due to comorbidities and diagnostic overlap with other common causes of vertigo (e.g., BPPV, Ménière’s disease), which may delay or obscure the diagnosis [40]. Biologically, VM is best explained by a central neural mechanism involving abnormal excitability and dysregulated interactions between migraine and vestibular networks across the brainstem, thalamus, and cortical processing areas. These interactions result in the characteristic co-occurrence of vertigo and migraine symptoms [40]. Supporting this model, preventive medications used for migraine that act on central pathways have demonstrated clinical benefit in VM, indicating that VM is driven primarily by central mechanisms rather than a peripheral vestibular disorder [34].

### 4.5. Clinical and Practical Implications

Our results have multiple clinical and practical implications. First, given its high prevalence, VM should be considered as the top differential diagnosis for recurrent vertigo in both pediatrics and adults, especially when accompanied by a family history of migraine or motion sickness. In adults, VM should be systematically differentiated from other vestibular disorders using standardized criteria. From a public health perspective, pediatric neurology, adult neurology, ENT, and primary care clinics should recognize VM as a common cause of recurrent vertigo; thus, it should be included along with their top differentials. Importantly, the consistent association with tinnitus across age groups underscores a potential gap in the current diagnostic criteria, which do not include this feature despite its documentation in the past literature. Early diagnosis has therapeutic benefits, as recent evidence suggests that preventive pharmacological and lifestyle changes may improve outcomes in both pediatric and adult patients, supporting the development of age-specific treatment pathways.

### 4.6. Strengths and Limitations

Our comprehensive review stands on a solid foundation as it represents the first review to assess VM prevalence and clinical features across age groups. By the inclusion of 21 studies from multiple countries, our results can be applicable to various populations. Subgroup analyses clarified age-related prevalence patterns, and integrating pediatric and adult studies enabled direct symptom comparison. The review protocol was registered on PROSPERO and followed PRISMA 2020 guidelines, ensuring methodological transparency and reproducibility.

Nevertheless, several limitations should be acknowledged. A major limitation observed is the high heterogeneity among studies, which may affect the reliability and applicability of our results. This is largely attributable to variability in diagnostic criteria, clinical settings, and study design. Most included studies were retrospective, and tertiary-based centers caused referral bias. Current diagnostic criteria may inadequately capture pediatric cases, leading to variability in prevalence estimates. Potential publication bias was suggested by funnel plot asymmetry.

### 4.7. Future Research

Prospective and experimental studies assessing long-term outcomes, treatment responsiveness, and quality of life in both pediatric and adult populations should be prioritized in future research. Beyond clinical diagnosis, the use of validated patient-reported outcome measures (PROMs) would offer a more comprehensive evaluation of disease burden and treatment response.

## 5. Conclusions

Vestibular migraine is one of the most common causes of vertigo across the lifespan, with a higher prevalence observed in pediatrics. The clinical presentation of VM varies between age groups, and usually, there is overlap between VM and other differentials of vertigo. Notably, variability in diagnostic criteria across studies represents a fundamental methodological challenge that substantially influences prevalence estimates and clinical presentation. These findings underscore the importance of heightened clinical awareness, age-adapted diagnostic criteria to facilitate early diagnosis, optimize prevention, and inform treatment across all age groups. In addition, large-scale, standardized, and longitudinal research is required to improve prevalence accuracy, evaluate pediatric-specific criteria, and assess long-term outcomes from childhood to adulthood.

## Figures and Tables

**Table 2:** Characteristics of pediatric studies on vestibular migraine, including diagnostic criteria, family history, clinical features, and episode duration.

Study ID	Age Range of Pediatric Patients	Diagnostic Criteria	Family History of Migraine	All Clinical Features	Most Common Clinical Feature Reported	Duration of Episodes
Jeyasakthy Saniasiaya, 2024 [14]	2–17 [GP]	The Barany Society	68.6% migraine	Imbalance, motion sickness (60%), clumsiness, developmental delay, speech/language delay	Vestibular and balance delay (for the general population, VM itself is not mentioned)	Not mentioned
Zülal Özdemir Uslu, 2024 [15]	1.5–17.9 [GP]	ICHD-3	In the general population, 165 (20.8%) patients, and more frequent in the VM group (49%)	Headache (36.4%), blackout (9.6%), nausea/vomiting (9.4%), syncope (5.1%), tinnitus (2.7%), photophobia (1.1%), staring (0.5%), convulsions (0.5%)	Headache 288 (36.4%)	Not mentioned
Thyra Langhagen, 2015 [8]	3–18 years [GP]	ICHD-3 beta and Bárány Society (adapted for children)	Positive in 65%	Headache (dVM 100%, pVM 82%, sVM 85%), Associated SV (17%, 42%, 35%), Abdominal pain (6%, 15%, 6%), Orthostatic dizziness (11%, 9%, 0%), Syncope (11%, 6%, 3%), Tinnitus (3%, 3%, 0%)	Headache (dVM 100%, pVM 82%, sVM 85%)	Not mentioned
Gedik-Soyuyuce et al., 2021 [16]	1–17 years (VM)	ICHD criteria	89% reported family history	Motion sickness (98%), family history (89%), transient positional nystagmus, spontaneous nystagmus, gaze-evoked nystagmus, and vertigo episodes	Motion sickness (98%)	Not reported
Swain et al., 2023 [21]	5–16 (VM)	Not specified	Family history of migraine and epilepsy documented but not quantified	Nausea/vomiting (87.17%), Headache (32.05%), Hearing impairment (10.25%), Tinnitus (8.97%), Fullness in ear (10.25%), Altered consciousness (2.56%), Visual impairment (2.56%), Diaphoresis (3.84%), URTI history (15.38%), Seizure (3.84%)	Nausea and vomiting (87.17%)	Seconds: 20.51%, Minutes: 42.30%, Hours: 26.92%, >24h: 10.25%
Carmona et al., 2018 [22]	1–18 (VM)	Barany Society & IHS 2012	100% of definite VM, 83% of probable VM	Vertigo ± nausea/vomiting, position change intolerance, photophobia, phonophobia, visual aura	Headache + vertigo (co-occurrence)	5 min to 72 h

Abbreviations: ICHD-3: International Classification of Headache Disorders, 3rd edition; VM: Vestibular Migraine; GP: General Population; dVM: Definite Vestibular Migraine; pVM: Probable Vestibular Migraine; sVM: Suspected Vestibular Migraine; URTI: Upper Respiratory Tract Infection; IHS: International Headache Society.

**Table 3:** Characteristics of adult studies on vestibular migraine, including diagnostic criteria, family history, clinical features, and episode duration.

Study ID	Age Range	Diagnostic Criteria	Family History of Migraine	All Clinical Features	Most Common Clinical Feature Reported	Duration of Episodes
Soo-Jin Cho, 2015 [11]	28–49.5 (VM), 19–100 (GP)	ICHD-3 beta appendix; Bárány & IHS consensus	Not reported	Head motion-induced dizziness + nausea (36.9%), spontaneous vertigo (26.2%), positional vertigo (21.5%), head motion vertigo (15.4%)	Headache (primary vs. secondary distribution described)	Vestibular: median 180 min (60–630); VM: 5–60 min (38.5%), 1–24 h (55.4%), 24–72 h (6.2%)
Roberto Teggi, 2018 [13]	12–50 [GP], 18–78 [VM]	Bárány Society + ICHD-3	67.4% (first/second-degree), 87.3% in aura (*p* = 0.001)	Headache + vertigo (synchronous), Phonophobia (54.5%), Photophobia (53.8%), Lightheadedness (21.1%), Blurred/Double vision (7.9%), Loss of vision (6.1%), Weakness (9.5%), Nausea (79.9%), Vomiting (29%)	Nausea (79.9%)	Headache: <2 h (6.2%), 3–4 h (15.5%), 5–12 h (19.8%), 12–24 h (37.6%), 1–3 d (20.8%), >1 w (0.1%)
Lin & Rauch, 2024 [17]	20–89 (VM)	Bárány + IHS (2012)	Not reported	Episodic vertigo with migrainous symptoms, photophobia, phonophobia, aura, and motion sickness	Vertigo/dizziness	5 min to 72 h
Çelebisoy et al., 2022 [12]	17–74 (VM)	ICHD-3 (2018)	Migraine: 72.5%, Vertigo: 29.2%	Nausea (60.7%), Vomiting (17.1%), Photophobia/Phonophobia (41.9%), Osmophobia (19.5%), Allodynia (12.3%), Tinnitus (40.5%), Aural fullness (32.3%)	Nausea (60.7%)	Vertigo: 5–60 min (24.1%), 60 m–24 h (68.7%), >24 h (7.2%); Headache: 4–24 h (76.4%), >24 h (23.6%)
Power et al., 2018 [18]	17–84 (VM only)	ICHD-3 beta + Bárány (neurologist dx)	22% family history	Vertigo (96%), Headache (60%), Visual disturbance (51%), Nausea (49%), Tinnitus (44%), Aural fullness (30%), Phonophobia (26%), Hearing loss (23%), Vomiting (19%), Neuro signs (14%), Otalgia (2%)	Vertigo (96%)	5 min to several days
Dessai et al., 2019 [19]	18–79 (GP)	ICHD-3 + Bárány (2013)	Not reported	Episodic vertigo, constant imbalance, positional vertigo, disequilibrium, light-headedness	Episodic vertigo	4 to 24 h
Teggi et al., 2018 [13]	19–76 (VM)	Bárány/ICHD criteria (definite VM)	Migraine 70.2%, Vertigo 66.3%, VM 21.4%, MD 7.1%	Internal vertigo (73%), external vertigo (25%), dizziness (47.2%), postural symptoms (61.5%)	Internal vertigo (73%), Nausea (59.9%), Photophobia (44%)	<5 m (23%), 6–60 m (21.8%), 1–4 h (11.5%), 5–24 h (17.5%), up to 3 d (5.5%), >3 d (2.8%)
Toriyama et al., 2021 [20]	20–85 (VM), 18–65 [GP]	ICHD-III beta + Bárány vestibular criteria	55.4% first-degree relatives	Vertigo, dizziness, unsteadiness	Nausea/vomiting (100%), Photophobia (92.3%), Allodynia (76.9%)	Not specified; mean migraine attack 24.4 ± 29.8 h
Zhang et al., 2015 [23]	23–67 (VM)	ICHD-III beta	36% positive family history	Spontaneous vertigo (85%), Positional vertigo (18%), Head motion (12%), Visual aura (13%), Photophobia (87%), Phonophobia (85%), Tinnitus (10%), Aural fullness (4%), Hearing loss (3%)	Photophobia (87%), Phonophobia (85%)	Seconds to days; 25% <5 m, 75% within 72 h

Abbreviations: ICHD-3: International Classification of Headache Disorders, 3rd edition; VM: Vestibular Migraine; GP: General Population; IHS: International Headache Society; MD: Ménière’s Disease.

**Table 4:** Prevalence of VM among pediatric patients across included cohorts, with proportions and standard errors.

Study ID (Author, Year)	VM Cases	Total	Prevalence (*p*)	SE
Alicia Wang, 2020 [24]	357	1021	0.3497 (34.97%)	0.0149
Juliana Duarte, 2020 [25]	19	117	0.1624 (16.24%)	0.0341
Hyo-Jung Kim, 2020 [26]	124	355	0.3493 (34.93%)	0.0253
Alana L. Ferreira, 2024 [27]	110	471	0.2335 (23.35%)	0.0195
Gedik-Soyuyuce et al., 2021 [16]	48	203	0.2365 (23.65%)	0.0298
Lee et al., 2017 [29]	120	411	0.2920 (29.20%)	0.0224
Swain et al., 2023 [21]	24	78	0.3077 (30.77%)	0.0523
Carmona et al., 2018 [22]	15	247	0.0607 (6.07%)	0.0152

Abbreviations: VM: Vestibular migraine, SE: Standard Error.

**Table 5:** Prevalence of VM among adult patients across included cohorts, with proportions and standard errors.

Study ID (Author, Year)	VM Cases	Total	Prevalence (*p*)	SE
Hyo-Jung Kim, 2020 [26]	2387	20,811	0.1147 (11.47%)	0.00221
Soo-Jin Cho, 2015 [11]	65	1414	0.0460 (4.60%)	0.00557
Alana L. Ferreira, 2024 [27]	69	1463	0.0472 (4.72%)	0.00554
Eric J. Formeister, 2018 [28]	584	21,781	0.0268 (2.68%)	0.00110
Lin & Rauch, 2024 [17]	204	1285	0.1588 (15.88%)	0.01020
Power et al., 2018 [18]	90	220	0.4091 (40.91%)	0.03315
Dessai et al., 2019 [19]	28	218	0.1284 (12.84%)	0.02266
Toriyama et al., 2021 [20]	65	245	0.2653 (26.53%)	0.02821

Abbreviations: VM: Vestibular migraine, SE: Standard Error.

**Table 6:** Prevalence of VM among patients presenting with vertigo, with calculated proportions and standard errors.

Study ID (Author, Year)	VM Cases	Total	Prevalence (*p*)	SE
Alicia Wang, 2020 [24]	357	1021	0.3497 (34.97%)	0.0149
Juliana Duarte, 2020 [25]	19	63	0.3016 (30.16%)	0.0578
Hyo-Jung Kim, 2020 [26]	2515	21,166	0.1188 (11.88%)	0.00222
Eric J. Formeister, 2018 [28]	584	2490	0.2345 (23.45%)	0.00849
Gedik-Soyuyuce et al., 2021 [16]	83	203	0.4089 (40.89%)	0.0345
Lee et al., 2017 [29]	120	411	0.2920 (29.20%)	0.0224
Lin & Rauch, 2024 [17]	204	1285	0.1588 (15.88%)	0.0102
Dessai et al., 2019 [19]	28	218	0.1284 (12.84%)	0.0227
Swain et al., 2023 [21]	24	78	0.3077 (30.77%)	0.0523
Carmona et al., 2018 [22]	76	247	0.3077 (30.77%)	0.0294

Abbreviations: VM: Vestibular migraine, SE: Standard Error.

## Data Availability

No new data were created or analyzed in this study. Data sharing is not applicable to this article.

## Abbreviations

The following abbreviations are used in this manuscript:

VM	Vestibular migraine
BPPV	Benign Paroxysmal Positional Vertigo
